# Some linguistic and pragmatic considerations affecting science reporting in English by non-native speakers of the language

**DOI:** 10.2478/v10102-012-0018-1

**Published:** 2012-06

**Authors:** Magda Kourilova-Urbanczik

**Affiliations:** 1 Institute of Experimental Pharmacology & Toxicology, Slovak Academy of Sciences, SK-84104 Bratislava, Slovakia

**Keywords:** English academic discourse, non-native speakers, language barrier

## Abstract

Approximately 50% of publications in English peer reviewed journals are contributed by non-native speakers (NNS) of the language. Basic thought processes are considered to be universal yet there are differences in thought patterns and particularly in discourse management of writers with different linguistic and cultural backgrounds. The study highlights some areas of potential incompatibility in native and NNS processing of English scientific papers. Principles and conventions in generating academic discourse are considered in terms of frequently occurring failures of NNS to meet expectations of editors, reviewers, and readers. Major problem areas concern organization and flow of information, principles of cohesion and clarity, cultural constraints, especially those of politeness and negotiability of ideas, and the complicated area of English modality pragmatics. The aim of the paper is to sensitize NN authors of English academic reports to problem areas of discourse processing which are stumbling blocks, often affecting acceptance of manuscripts. The problems discussed are essential for acquiring pragmalinguistic and sociocultural competence in producing effective communication.

## Introduction

English has become the international language of science reporting both between native and non-native speakers (NNS) of the language and between NNS of different languages. In the life sciences, practically all the best peer-reviewed journals appear in English and at least 50% of the publications are contributed by NNS (Benfield & Feak, 2006). Though a remarkably high proportion (83.6%) of NNS academics (N=300) were reported to agree on the need for a single international language, 96.6% think that the dominance of English gives an advantage to native speakers (Ferguson *et al.*, 2011). Benfield & Howard (2000) speak of the language burden faced by English second language scientists.

Mistakes can be corrected, yet the English feel, a phenomenon that defies definition, is very hard to achieve. The belief that mastery of vocabulary and syntax results in successful communication is mistaken. Misinterpretation of messages is to a great extent due to the NNS's pragmatic failure, *i.e.* lack of ability to grasp how resources of a language code are put to use in the production of scientific discourse. The language code is highly systematic, well established and relatively easy to learn. Yet the principles, strategies and conventions that govern the use of the code in producing actual messages and discourse resulting in communicative success are hard to classify, systematize and acquire.

Though discourse features operate mostly by universal principles, there are cross-culturally different perceptions of appropriate language behavior. NNS often transfer discoursal patterns typical of their own language and culture but alien to English academic writing. Rather than imperfect grammatical competence, it is the NNS’ lack of insight into the pragmatics of interactive functions of the English language which accounts to a considerable extent for the failure of applying rhetorical strategies, syntactic and referential devices associated with the Anglo-American notions of objectivity and politeness in science reporting (Hinkel, 1999).

The sense in which scientific thought and thought processing is culture bound is on a more or less subconscious level. Social identities become manifest in terms of beliefs, values, behavior. Explicitly defined and described models are hard to find. We do not know where all the differences in discourse processing lie, and moreover, these undergo constant shifts in time. Research into the pragmatics of English as an international language is still very much in its initial phase. The findings available to date result from personal experience and research on fairly limited databases (Seidlhofer, 2003).

The most pronounced differences between native and NNS discourse concern the rhetoric of science reporting, namely the principles and conventions that would meet the native speaker's expectations of effectively and readily processed communication. NNS often transfer discourse patterns typical of their own language and culture but alien to English science reporting. Areas of failure concern the perspective and development of the theme, local and global organization of discourse, and particularly the rather complicated field of modality pragmatics. The latter includes among others negotiating procedures, pragmatic inference and a number of factors giving unusual communicative values and functions to grammatical structures. A broad range of devices for mastering rhetorical strategies is available in English, yet NNS possess but a very limited stock, and even this is not always used appropriately.

Based on decades of experience in teaching medical English, correcting scientific manuscripts written by NNS of English, and in assessing peer reviews, the presented contribution will try to identify some conventions of the endonormative model of scientific English writing.

Though in many areas of language usage there has been a long-lasting vigorous debate whether and which NNS deviations should be acceptable in lingua franca English (Widdowson, 1994; Perez-Llantada, 2007; Fiedler, 2011), in scientific manuscripts submitted to English, and particularly to British journals, we have to realize and accept that norms of correctness and appropriacy are to be derived from those applied in the native speaker country. On failing to do so, one has to expect harsh criticism and even rejection by reviewers and editors. The mean acceptance rate of manuscripts was reported to be 78% for native speakers and 49% for NNS authors (Benfield & Howard, 2000).

It is but a small solace to learn that native speakers appear to share some of the failure of writing readily comprehensible expository discourse, as reported by Ludbrook (2007) who considers the quality of English prose in biomedical scientific discourse to be deteriorating. His opinion is corroborated by an analysis of articles in Nature and Science published over the periods 1930–1990 and 1990–2000 characterizing the articles to be progressively more difficult to read (Knight, 2003), published under the rather pejorative title:'Clear as Mud’.

In the present paper, however, we are not concerned with native speaker shortcomings in producing intelligible English scientific papers. Our aim is to point out features that create problems specifically to NNS of English in writing acceptable manuscripts concerned with biomedical sciences.

## Some discourse features causing problems in NNS science reporting

### 1. Linear discourse development and paragraphing strategies

Conventions of Anglo-American composition require rational argumentation, objectivity in the writer's position and views, factuality in justification and proof, mitigation of own claims and politeness in assessing claims of other authors. Further, the English native speaker expects a linear development in the sequence of thought processing. A coherent informative text should have a clear structure which spells out the main idea and subtopics as well as the hierarchy of their importance.

The thought organizing structure of scientific journal articles, namely the IMRAD procedure (Introduction, Material and Methods, Results, and Discussion) is an invaluable help in thought processing. Nevertheless, NNS discourse has often been criticized as giving an un-English feel. Digressions and unjustified repetitions in handling the author's argument and preference for indirection have been pointed out where linear discourse development is expected. NNS tend to present a whole range of potential circumstances, while at the same time adequate evidence and qualifying support for relevant issues is lacking. One reviewer expressed his criticism rather bluntly: ‘This paragraph is unduly long and yet it screams for explanation.’

Digressions are more frequent in Slovak, German, or French than in English science reporting. Particularly German discourse has been often criticized as unreadable due to the difficulty in distilling the topic from an intricate network of parenthetical amplifications resulting in long sentences and paragraphs.

In several instances of NNS writing, the discourse is organized around the data rather than reasoning. Moreover, in their attempts to construct a unified idea flow, NNS rely only on a limited repertoire of accessible linguistic means. In English the main argument is developed throughout the whole discourse in a linear fashion with appropriate cohesion devices and sentence transitions so that the reader has no difficulty in following the line of thought and identifying the developing stages of the argument (Hinkel, 2001).

In mastering the logical flow of thought, different languages and cultures apply different paragraphing strategies. Literary paragraphs with their broad range of functions can be shaped by various approaches. The English expository paragraph, however, has to adhere to the established pattern. Each paragraph is a logical unit of thought. It develops a new idea and marks a new stage in the author's argument. The most important sentences in a paragraph are the first and last one. The first should provide a connection with the information that has gone before, the last should emphasize new information. English further operates with the excellent device of physical and conceptual paragraphs, where each physical paragraph deals with a different logical focus yet two or three of them may constitute a conceptual paragraph which is one logical unit. This is an excellent organizational device promoting quick understanding.

### 2. Sentence Perspective and Word Order

One of the major hurdles for the non-native writer of English discourse is the question of word order. Word order can influence meaning in subtle ways and so form part of the discourse structure. The initial constituent of a clause (a group of words containing a subject and a finite word and forming part of a complex sentence) is called the theme and is the starting point of the message. It is chosen from known or given information, while the new item forms the climax, the focus of the statement, and receives the important end position.

The complex notion of the word order is an area where English is less flexible than many other languages. In Slovak *e.g.*, there is no obligation to express the grammatical subject and due to the rich system of inflections, the movement of sentence constituents is fairly free of grammatical constrictions. Focalizing can be achieved by simple rearrangement of the constituents. In English word order indicates grammatical relationships. The movement of a given semantic element requires assignment of a new syntactic function to the semantic unit in question and a corresponding change in the syntactic structure. Thus *e.g.* the initial subject-agent of an active sentence achieves the final position only by passive transformation. (Example: ‘The effort to indicate unambiguously the information focus of the sentence motivates the syntactic choices in writing’ ‘The syntactic choices in writing are motivated by the effort to indicate unambiguously the information focus’). The passive voice is frequently used to ensure that the focus falls on a prominent element. The choice of the active or passive form is not arbitrary and depends on the context and the author's intentions.

As mentioned above, the information which relates to the preceding text comes first, while what the author intends to focalize is to be found at the end, characterized by the well-known end-weight of the English sentence. (Example: ‘The animals were divided into two groups (…). Decreased saturation of oxygen caused **in both groups** decrease of…..’) ‘In both groups’ is clearly the theme, the already mentioned item, and should be placed at the beginning of the second sentence, and moreover, the focalized object would thus closely follow the subject. Another of the abundant examples where NNS fail to put the actual theme into the frontal position: ‘SERCA dysfunction **in the above mentioned pathological states** was attributed to excessive production of…’. The sentence should run as follows: ‘In the above mentioned pathological states, SERCA dysfunction was attributed to….’.

Spoken language, with its prosodic clues (placement of the intonation nucleus), has an excellent device for indicating the information focus without any change in word order. Written language has to employ variation in the word order to highlight the focus of information and thus to achieve communicative dynamism. The writer must construct clauses and whole sentences with careful word order, punctuation and discourse implication about what is considered given or new information to make up for the loss of such explicit markers of emphasis as stress and pitch in speech. The thematic (initial) position and the focal (final) position are central for interpreting and integrating the message of a clause. Compared to spoken language, there is a relatively greater complexity of structure and more constituents are complementing the verb. This results in greater competition for the individual positions in the sentence and a marked word-order in academic discourse (Kies, 1990; Rohrauer & Dubec, 2011).

NNS have been found to violate even the basic rule that the subject has to be in the preverbal position while all constituents are normally postverbal and that subject and verb have to be kept close together. This may be partly under the influence of the exceptional postverbal position of the subject in the‘There is/are …’ construction, yet native language transfer is a more likely cause. Further, NNS appear to have problems with adverbs and adverbial phrases (*e.g.* ‘Over the last decade, … ‘ ‘In our recent study …’) by putting them anywhere in the sentence instead of fronting them or giving them the final position. Another frequent deviation is the position of the past participle. Learners of English are trained to put the modifier before the noun (*e.g.* normal activities, direct measurements). In written discourse, however, the past participle as modifier is placed **after** the noun when there is a reference to some previous information in the text (*e.g.* the animals studied, the method applied, the substances tested).

Focalizing and the principle of end-weight provide important consequences for the semantic interpretation of a sentence. It is not uncommon to find examples of deviant word-order in native speaker scientific discourse. The adjective may be fronted for the sake of emphasis ( *e.g.* ‘Very important is the screening …’ ‘More optimistically, Hart *et al.* (2010) showed that good control was correlated with..’).‘Optimistically’ links the sentence to what was said before in the text and introduces an element of contrast. For the NN writer it is not easy to decide when a deviant word order is justified and when unacceptable as it signals the influence of native language interference.

### 3. Parataxis and hypotaxis – main and subordinate clauses

There are different ways of smuggling in propositions without stating them. Thus the use or absence of subordination carries important signals for interpreting and integrating messages.

Hypotaxis (syntactic subordination, the use of main and subordinate clauses) allows for hierarchical arrangement of themes and foci to foreground and background information in clauses. An appropriate use of syntactic subordination can yield emphasis and imply the author's attitude and his/her evaluation of facts, data, findings. It provides focus and prominence to important discourse entities and creates a differential hierarchical discourse structure signaling important communicative values by backgrounding presupposed or known information through the themes and foregrounding new, unpredictable information through end-focus, facilitating comprehension.

Parataxis, a sequence of main clauses without subordination and connectors, allows for multiple themes and foci in a sequence of clauses where no one clause dominates another. Parataxis leaves more to infer and is sometimes used in peer writing where producer of information and recipient share a large amount of background knowledge and possess inferencing capacities. For NNS, who often lack this competence, parataxis may cause incoherence and loss of important implications. And yet, as producers of information, NNS present a too scanty use of subordination, which may create an undifferentiated, non-hierarchical structure, failing native speaker expectations. The difference between degrees of subordination in native and NNS discourse has repeatedly been found significant. Thus *e.g.* 25 hypotactic structures occurred per 1000 words in native speaker scientific communication compared to only 4 used by NNS (Kourilova, 1992). Golebiowski (2006) found NNS to use parataxis in 32% compared to 15% in native speaker texts.

Native speakers tend to indicate more readily the distribution of functional weight of their messages by using hypotactic structures, while NNS leave readers to their own resources in the selection of information mandatory for discourse comprehension. But not only deficient but also inadequate use of subordination may create incomprehensibility by highlighting wrong bits of information and assigning unsubstantiated pragmatic force to utterances. Proper clause structure reflects the academic quality of written expository discourse.

#### 3.1. Defining and nondefining relative clauses

The different management of relative clauses in English and in other languages, *e.g.* Slovak, is a further problem area. While Slovak relative clauses are virtually all separated by commas from the main clause, in English the nondefining is separated, while the defining is without commas. In English implicit meanings can be involved in the use of relative clauses and in the very choice between a defining and nondefining clause. In many instances it is however the proposition that determines the choice. Consider the difference between the two sentences: ‘TSH, which is produced in the pituitary, stimulates the thyroid gland’ and ‘TSH which is produced in the pituitary stimulates the thyroid gland.’ While the nondefining relative clause, separated by commas, implies that the pituitary is the only source of TSH, which is not true, the second example, the defining relative clause without commas, leaves open the possibility that TSH is produced also elsewhere, *i.e.* in the periphery. Or:'The boys, who took the bus, arrived on time’ means that all the boys took the bus, while ‘The boys who took the bus arrived on time’ means that only those arrived on time who took the bus.

In many instances, however, it is the prospective receiver of information whose anticipated background knowledge determines not only the depth and density of information but also modality pragmatics, including the choice between defining and nondefining relative clauses. When addressing a nonexpert, *e.g.* in textbooks, the majority of information must be considered new and unknown to the processor and is thus rather frequently presented in the form of defining clauses. When however experts are addressed in scientific journals, much shared background knowledge can be expected to be supplied by the processor and information is presented in the form of nondefining clauses. In this communicative situation, the defining relative clause may be considered too explanatory and thus even arrogant and impolite. Yet not only the prospective reader but also the whole discourse, the context, may influence the choice. An issue considered new to the reader would be expressed by a defining relative clause. When the writer returns to this issue in the text, and thus may consider it to be already known to the reader, practically the same proposition would now appear in a nondefining clause. Though this practice is logical, it may appear somewhat confusing to NNS.

On investigating the rate of defining and nondefining relative clauses in a corpus of 50 000 textbook words, 840 relative clauses were found. Of these 78.6% were defining and 21.4% were nondefining. This ratio was practically reversed in the corpus of 50 000 words of scientific journal articles. Of the 610 relative clauses recorded only 18.6% were defining and 81.4% were nondefining, highlighting the importance of the prospective processor of information with shared background knowledge (Kourilova, 1992).

### 4. Modality and communicative competence

The processing of objective fact and subjective evaluation is the crucial task of science reporting. This implies that statements contain personal attitudes and claims based on plausible reasoning. The writer's commitment to the truth value of a proposition, qualifying it as to the degree in the continuum extending from possibility, probability to certainty, from specificity to generality, variance to invariance, remoteness to relevance, is expressed or implied by means of the modality system. The markers of this system also provide flexibility in projecting different shades of the writer's modesty, caution and politeness towards the scientific community.

Modality is a very complex phenomenon, which derives its devices from practically all grammatical categories, syntactical and organizational means (including also some of the above mentioned categories, such as word order and subordination) and a broad range of lexical items.

Both producers and receivers of information need insight into this linguistic and socio-cultural problem area to prevent messages from being misunderstood, misinterpreted, judged untruthful, uninformative, impolite or even lost. Modality, more than any other language phenomenon, depends on context (the discourse as a whole) and on cotext (the communicative situation, *e.g.* peer writing with many implied messages vs. textbooks requiring a high degree of explicitness). One could hardly speak of rules, there are rather modality strategies and conventions, yet these play a crucial role in determining the intended meaning.

#### 4.1. Tentatively expressed opinion – hedges

In manuscripts written by NNS of English there is much more straightforward representation of knowledge, high assertiveness and seeming disregard to the scientific community expressed in unmodified sentences. It is the NNS's insensitivity or lack of knowledge of discourse features manifested through the intricate English system of modality that makes their discourse appear overconfident and unjustifiably conclusive. This may be due to a wrong use of extratemporal values of tenses, neglect of the communicative situation in passivization, to the use of defining and non-defining relative clauses, to lack of insight into the generalizing value of the article, as well as to the inability to master politeness markers as an integral part of the English cultural system.

Both in spoken and written native speaker discourse there is a preference for tentative rather than assertive expressions of opinion and evaluation achieved by **hedges**, devices that mark a statement as provisional and not generalized. Hedges play a critical role in gaining ratification for claims from a powerful peer group by allowing writers to present statements with appropriate accuracy, caution, and humility, expressing possibility rather than certainty and overconfidence. It is impossible to avoid hedges on the long way before a hypothesis becomes conclusively established and accepted by the scientific community (Hyland, 1998).

Politeness strategies are used to mitigate communicative acts that create tension or are perceived as offending, face threatening. This applies particularly to claims and negative judgement expressed in scientific journal articles. Thus *e.g.* in the statement ‘The review appears to be somewhat sketchy…’ criticism is strongly mitigated by the combination of impersonalization and double hedging (*i.e.* ‘appears’ and ‘somewhat’). An assertive and offending statement would run as follows: ‘I find the review to be rather sketchy.’ It is to be realized that the combination of personalization with an assertive verb is impolite as it does not leave the proposition open to negotiation by the scientific community, as *e.g.* ‘We demonstrated ….’ or the practically unacceptable version with the present perfect: ‘We have demonstrated…’. Whereas ‘The results show…’ or even more mitigated, in decreasing order of assertiveness: ‘The results indicate… ‘, ‘The results suggest…’, ‘The results appear to suggest…’ give ample space for negotiation.

Hedging should not be overdone, as *e.g.* in ‘One may tentatively suggest… ’ with impersonalization and three hedges. Redundancy of hedges implies mostly vagueness rather than politeness and weak claims do not deserve to be published.

When however the claims are fairly strong yet the obtained results fail to meet the expectations, pessimism is permitted, as *e.g.* in ’… the results failed to elucidate fully…’, ‘… as yet it must be regarded unproved…’, ‘…the results do not exclude the possibility…’, or the overused ‘… further studies are required to prove…’. Such implied disappointment addresses particularly editors and referees who tend to dismiss negative findings though they may be signposts for continuing the quest, inviting other researchers to share the credit.

In a corpus of 22800 words of scientific discourse written by native speakers, the number of hedges per100 words amounted to 6.5 in the interpretive sections Introduction and Discussion, while in the factual, reporting sections the number of hedges per 100 words was only 0.05. In NNS scientific communication there were only 2.1 hedges per 100 words in the interpretive sections of a corpus of 21200 words. Up to 80% of the hedges used concerned only the modal verb ‘may’, neglecting all the other means available (Kourilova, 1995). The need to hedge propositions and claims to show appropriate amount of hesitation and politeness is a feature specific to Anglo-American (and particularly to British) rhetorical tradition.

Of the great gamut of devices helping to create tentative, mitigated language in scientific journal articles let me treat at least some instances that may signal the NNS's sociopragmatic competence, namely:

extratemporal value of tenses, use of the article, impersonalization, and the broad gamut of lexical items.

#### 4.2. Extratemporal Values of Tenses

A journal article is a live model of actual language in use. Yet scientific discourse has a semiautonomous position so that many grammatical structures may be assigned functions characteristic only of a given communicative situation. It is then the unfamiliarity with modality conventions which govern the use of familiar structures that accounts for deficient communication and may even appear offensive.

The use of tenses has traditionally been explained from the aspect of real time. The availability of this relationship is however rather limited in English scientific discourse. It is the context and the communicative situation including the author's subjective attitudes and intentions that strongly affect the functional value of tenses. Writers can manipulate the temporal identity of actions, events, aspects of the research process so as to present their own perspective and purposes in implying the degree of relevance, certainty, generality, and caution. It is the author's decision to refer to an event or action as timeless generalization or a timebound result.

In the excellent thought organizing IMRAD (Introduction, Materials and methods, Results, Discussion) pattern of scientific papers, different conventions apply to individual sections.

In the Introduction and Discussion sections, the use of the **Present Simple** is rather rare. It indicates absolutely and generally accepted truth value and is never used in reference to the author's own work or findings. It may but is rarely used to indicate the author's intention of distance to a cited paper. There are only few NNS researchers who would understand the implicit value of the present tense in the Introduction referring to a literary source as the author's signal of distance without comment (*e.g.* ‘Casey (2010) reports positive responses’). The implicit message is: ‘The findings are absolutely unreliable and do not even deserve to be commented or discussed.’ This rhetorical signal is however situation-bound and applies only to the Introduction of a journal article and not to the Discussion section.

The use of the **Present Perfect** (*e.g.* Smith *et al.* have reported) signals highly accepted validity of other authors’ finding or hypothesis, yet only as to the present state of the art and no commitment with respect to future findings. It can further indicate a very important feature, *i.e.* high relevance to the present paper and in the Introduction also the author's promise of continued attention and future comment.

The Present Perfect tense is however used only sparingly and its strategic choice exerts the function of implicit assessment of literary data both in the Introduction and Discussion sections of scientific journal articles. It should not be used in reference to the author's own findings. Thus the sentence: ‘In contrast to Wang *et al.* (2008) we have found …’ is face threatening and therefore unacceptable.

Exceptionally, the aspect of importance may override the rule which prevents the use of the present perfect tense with an explicit indicator of the past. Thus *e.g.* the sentence ‘In 1978 Axelrod has established the structure of ACTH’ written by a native speaker is not poor grammar. It indicates the exceptional importance of the finding also for the present time.

The **Simple Past** is neutral and practically obligatory in reference to the author's own work and findings. When citing other authors, it is mostly neutral, yet it may refer to a not confirmed or refuted hypothesis. ‘The disease was reported to affect mostly women’ can be neutral or it may signal doubt, whereas ‘The disease has been reported to affect mostly women’ indicates acceptance of the finding. Lack of proof of other authors’ findings can be strongly signaled by the Simple Past in the ‘if’ clause and Present Simple in the main clause (*e.g.* ‘If this applied also to other species, it is a remarkable finding’), clearly indicating that substantial evidence is still lacking. In combination with an expression grammatically requiring the Present Perfect (*e.g.* so far, to date), the Simple Past strongly indicates not only shortcoming but irrelevance to the present search for truth, as *e.g.*'The single cohort study which was published so far investigated a statistically nonrepresentative series of patients’. Relevance may become more important than the factor of real time and grammatical rules are subordinated to modality purposes.

Boundaries of tenses separated by modality considerations can be rather fluid. The choice of an atypical tense for a given objective degree of distance is acceptable when the author wants to give the subjective impression of remoteness from the present moment. Thus *e.g.* in the statement ‘His finding was irreproducible’, the choice of the Simple Past implies a subjective degree of remoteness from the present time. The finding is still irreproducible and the verb should be in the present tense, yet the author intends to indicate remoteness in terms of something being absolutely false and thus irrelevant for the present search of truth. Relevance overrides the factor of real time.

When situational factors come into play, namely when there is reference to the given paper, the Present Simple is a must: (‘The obtained findings **are** summarized as follows…’;

′Fig. 4 **shows** the relationship…′).

The language associated with graphs and tables underlies different conventions. The Present Simple is practically prevailing.

In contrast to the two commenting sections (Introduction and Discussion), virtually no strategic choices are operative in the reporting sections Material and Methods and Results, with the predominant use of the **Simple Past** and heavy passivization. These sections provide statements of facts and stand outside the system of modality. Yet it has to be pointed out that considered from the discourse angle operative in the commenting sections, a statement reported as a finding in the reporting sections falls within the system of epistemic modality when pesented and discussed in the commenting sections. In the latter parts of the paper findings have to be handled cautiously as potential facts and no categorical truth value is to be declared by the author. All findings have first to be accepted and acknowledged by the scientific community.

Every scientific report makes a claim, as only this justifies its publication. A claim, however, involves tension and may threaten or deny claims of other researchers. Thus claims must be mitigated by epistemic devices of politeness. In English science reporting there is indeed a rather strict constraint in deciding on the value of your own findings and that also in cases when you are convinced of the uniqueness of your results. This applies also to claims of Nobel prize value. Watson and Crick started the report on their revolutionary discovery of the DNA structure modestly with the words ‘We wish to suggest…’. Modesty is expected from the author and it is up to the scientific community to decide on the validity and generality ranking of a contribution.

#### 4.3. Pragmatic values of the article

Complete insight into the broad and rather complicated area of pragmatic values of the article remains unattainable for a considerable majority of NNS. And yet, the article or its absence may be an important modality device and whole books have been devoted to its use. In their error analysis of NNS manuscripts, Benfield and Howard (2000) reported that the incorrect or missing use of the article constituted the largest number of errors, followed by ungrammatical sentence structure.

There is a great difference between the sentence: ‘We investigated **the** mechanisms involved in free radical induced damage’ or the same sentence in which the article is omitted. With the use of the definite article you declare that all the mechanisms in question were investigated. On omitting the article, you leave open the possibility that other mechanisms, besides those you studied, may also be operative. In the sentence: 'These disturbances result in an imbalance of hormonal factors and **a/the** subsequent development of autoimmune disease’ the definite article indicates an unavoidable outcome.

It appears worth mentioning that the definite article with a noun in singular indicates general representation. For instance: ‘Inhibition of cholinesterase activity ….in the rat’ concerns the species ‘rat’. ‘Effect of …on the rabbit aorta’ refers to aortas in the species ‘rabbit’. Or: ‘the failing heart’ applies to any heart in this condition. The only exception to this rule is ‘man’. ‘Man’ as species is used without the article. ‘Assessment of … in man’, or preferably ‘in humans’. ‘The man’ refers to one individual man.

In titles of scientific papers, the use of the article is rather restricted: ‘Safety of inhaled corticosteroids…’ ‘Effect of thyroid hormone on …’ and not ‘The safety of…’ and ‘The effect of …’, which in the text would however be correct.

The modality function of the article signaling high commitment to generality or absoluteness may make the writer appear overconfident. In peer reviews I found strong criticism of the improper use of the article indicating an inadequate level of commitment to the truth value of propositions, expressed as *e.g.* ‘You are not entitled to generalize’ or ‘The evidence presented falls short of justifying the use of the definite article’ (Kourilova, 1998).

#### 4.4. Personalization vs impersonalization

An important area where modality exerts its functions is the challenging of other authors’ claims. Agreement may be personalized yet disagreement should always be impersonalized and the author of the denied statement given in parentheses: ‘The increase was claimed to be induced by the release of … (Smith *et al.*, 2009). Impersonal phrasing prepares the reader for denial of views and approaches. Rival claims may be treated from zero agreement, always impersonalized (‘The data failed to establish…’) to mitigated disagreement (‘The mechanism is not likely to be operative under conditions of…’), or by commented refutation through grudging concession (‘Admittedly, considering the possibility…’). Weak agreement should be impersonalized (‘The findings may indicate….’), while full agreement can be personalized (‘His findings showed …’). When personal attribution is inserted in disagreement with rival claims (‘Smith claims…’) the denial is not only strong but also impolite and is but exceptionally used by native speakers of English.

Since direct criticism is practically inadmissible, many implicit or indirect ways can be used, as *e.g.* ‘Blood sampling by indwelling catheters excludes interference of nonspecific stress factors’, implying that the mentioned author's results are unreliable since he used an inappropriate method of blood sampling. Many uses of the passive voice can be pragmatic and can function as hedges (‘The finding was not corroborated by animal studies.’). The passive often serves as a typical marker of detached style of Anglo-American academic writing. The agentless passive can be employed to front thematic information and remove the agent from the prominent sentence position.

Nominalization may also be used as negative politeness in criticism (‘His addressing the problem failed to…’ or ‘His work is said to be the first attempt…’). The latter example is actually an only little mitigated denial of a priority claim. Rather intricate interactional problems are involved in dealing with priority claims. Independent claims must be credited, yet the credit may be somewhat mitigated, *e.g.*‘It has not escaped our notice that a similar mechanism was postulated’. The worst criticism of a rival claim is to ignore it. To be cited, even critically, has become a survival necessity for researchers.

#### 4.5. Lexical devices

Lexical signals of modality function pragmatically as markers of probability and certainty, specificity and generality, closeness and remoteness, as well as markers of politeness. Considering the broad repertoire of forms and structures which may express modality, and particularly their polysemic nature, it is practically impossible to establish a straightforward matrix into which all instances could be neatly fitted. Within the scope of the present paper and in accordance with its main aim, only some instances of frequently occurring NNS deviations will be mentioned.

Attention should be paid to two verbs in particular: ‘to suggest’ and ‘to demonstrate’. With ‘suggest’ great distance is signaled when used in the Simple Past. ‘The disease was suggested to affect mostly women’ indicates doubt or even disagreement, while ‘The disease has been suggested to affect mostly women’ means that though still at a hypothetical level, the suggestion is most probably true. This is an excellent means to signal accord with or to distance oneself from statements of other authors, rarely observed by NNS of English.

While the latter instance results ‘only’ in misinterpreted or lost message, the NNS’ use of ‘demonstrate’ referring to the author's own findings in the Simple Past can be perceived as overconfident and in the Present Perfect even as offensive. Not many Slovak writers are aware of the different force this verb has in their language (meaning mostly ‘to show’) and in English with the connotation ‘to prove, establish evidence’. This feature of cross-linguistic semantics can induce severe misunderstanding. One reviewer put his criticism of the phrase used by the author ‘we have clearly demonstrated’ rather bluntly: 'How dare you refer to your own results in this cocksure way!’ Instead of this excessive assertiveness, the use of the verbs ‘show’ or ‘indicate’ would have been appropriate. The use of the Present Perfect and the strong modifier ‘clearly’ made this statement culturally unacceptable in a scientific paper.

As expected, modal auxiliaries have the highest incidence of lexical items in signaling epistemic modality, with ‘may’ occupying the first rank. This is particularly true of manuscripts of NNS who frequently lack control of other means expressing modality.

The implicit measure of truth associated with a statement can be signaled in a decreasing scale from certainty to low possibility as follows:

1. **full verb in the present tense** – categorical certainty: ‘In the given concentration, the substance elicits adverse effects’.
2. **do/does** – boldly stressed certainty that has been or is expected to be questioned (not to be recommended for NNS manuscripts as it may appear face threatening): ‘In the given concentration the substance does elicit adverse effects’.
3. **must** – high degree of confidence: ‘In the given concentration, the substance must elicit adverse effects’ (strong argumentation).
4. **should/ought to** – somewhat lower but still high degree of confidence: ‘In the given concentration, the substance should elicit adverse effects’.
5. **may** – possibility signaling willingness to speculate on the probability of the event: ‘In the given concentration, the substance may elicit adverse effects’. Your judgement is based on some evidence.
6. **can** – tentative probability: ‘In the given concentration, the substance can elicit adverse effects’ (should be avoided since there may be an overlap into ‘can’ expressing ability).
7. **might/could** – low possibility with caution: ‘In the given concentration, the substance might elicit adverse effects’.
8. **must** – to be differentiated from /3/ – author's personal opinion not based on evidence: ‘In the given concentration, the substance **must** elicit adverse effects’ (it expresses virtual certainty and should not be used in science reporting).

Lexical means that suggest absolutes on the scale of likelihood do not appear to create problems, yet it is practically impossible to give precise delimitations to the scope of non-absolute concepts. This does not apply only to modal auxiliaries but also to other means of expressing modality, which appear to present even greater hurdles. Thus NNS show a reluctance to use adverbs of all kinds and modal adverbs in particular.

The adverbs given below express degrees of likelihood emanating from the writer's personal orientation towards the proposition, often mitigated out of politeness to rivals or the scientific community as such.

**Certainty:** definitely**,** undoubtedly**,** unquestionably**,** undeniably, clearly, evidently, obviously

**Probability:** arguably, assumably (based on evidence), presumably (based on speculation), reportedly, apparently (caution – the last adverb has two practically opposite meanings: ‘evidently’ – signaling certainty, and ‘seemingly’ – not necessarily true).

**Possibility:** conceivably, perhaps, maybe.

Adverbs of this kind may appear in one of three positions: initially, after the subject and before the verb, or immediately after the verb:

**Obviously,** in the concentration used, the substance elicits adverse effects.

In the concentration used, the substance **obviously** elicits adverse effects.

In the concentration used, the substance elicits **obviously** adverse effects.

There are however several other forms which may express comment on the truth value of a proposition, as *e.g.* nouns, adjectives, adjectival phrases, *etc.* Moreover, all these devices can be used with intensifiers, such as ‘very, most, highly, extremely, absolutely’ or the often inappropriately used RATHER, which can express different meanings depending on the adjectives and adverbs it is associated with. Thus:

- with unfavorable adjectives and adverbs it means: ‘moderately, to some extent, quite’,while ‘fairly’ has the same meaning with favorable adjectives. EXAMPLE: ‘His condition was rather (=quite) serious on admission but after anticonvulsive treatment he did fairly (=quite) well.’
- With favorable adjectives and adverbs it means ‘very’ and is definitely more positive than ‘fairly’. EXAMPLE: ‘Your paper was rather good’ (= great compliment). ‘His English is fairly good’ (= quite good but leaves much to be desired).
- With adjectives and adverbs which are neither favorable nor unfavorable ‘rather’ expresses disapproval. EXAMPLE: ‘The Introduction is rather long.’ (=in my opinion it is too long). ‘The Introduction is fairly long.’ (=it appears to meet the requirement). The choice here is psychological rather than grammatical.
- With comparatives, ‘rather’ signals something less than expected, somewhat, a little, a bit. EXAMPLE: ‘The level was rather higher than expected.’ ‘The outcome was rather more complicated.’ (= we expected a little lower level and a somewhat less complicated outcome).
- I'd rather + past tense expresses a wish. EXAMPLE: ‘I'd rather you finished reading the article before commenting on it.’

Caution is well advised particularly on reporting on the author's own findings and can be signaled by the very useful group of mitigators, such as ‘almost, practically, virtually, somewhat, to a certain extent/degree, hardly, barely,*etc.* Mitigators signaling limitation are important epistemic devices particularly in biomedical sciences, where extrapolation and generalization carry a high risk of misconception. Limitation can be marked by phrases as ‘for the time being, in the species studied, only if, under the given conditions’, *etc.*

The above is but a sketchy indication of the relative power of devices expressing modality. The correspondences between the items mentioned are not particularly clear-cut. There is a certain amount of vagueness and overlap. In order to make a decision on an appropriate choice of the means of modality continual contextualization is necessary.

This applies particularly to **discourse markers**, called also **connectors, conjuncts**, which are logical signals for generating interconnected series of sentences in an integrated text. They tell us how ideas are organized and how sentences relate. To be aware of plane changes and to mark them appropriately is an important subskill in efficiently organizing main ideas, supporting points and supplementary information.

Conjuncts can be divided into several groups as additive, causal, temporal, paraphrasing, explicative, summarizing, which should not present considerable problems. The conditional conjunct UNLESS, however, appears to be difficult to handle and it is practically absent in NNS manuscripts. EXAMPLES: 1) ‘A virus lacks the tools to reproduce unless it invades a living cell.’ 2) ‘We can't operate on you unless you sign the informed consent.’ 1) + 2) A will not happen if it is not preceded by B. 3) ‘We can speak of improvement, unless the vomiting starts again.’ 3) A will happen if it is not stopped by B. ‘Unless’ can be substituted by ‘if not’, ‘except when’, ‘except that’.

A further group of conjuncts, *i.e.* the markers of dissonance, create many problems and much misunderstanding (Borkin, 1979). They are the most versatile in their functions and can hardly be understood without taking into consideration the discourse contexts in which they appear, the chain of reasoning and line of argumentation developed by the author. In light of this crucial implication, only very limited explanation of the semantics and pragmatics of some of these important discourse markers can be offered within the frame of this presentation.

In general, dissonant conjuncts mark the introduction of a statement or group of statements that is viewed as in some sense in conflict with a prior statement or group of statements. The dissonance can be of empirical nature, *i.e.* dissonance based on knowledge of the state of the art, and thus outside the text, and of rhetorical, text-internal nature, *i.e.* dissonance related to the ongoing process of argumentation, evaluation, deliberation, or debate, based on communicative purposes of the author.

The conjunct YET marks seeming incompatibility or implied conflict of empirical nature. Since the degree of implicitness is very high in peer writing, the inference is not only left unstated but is understandable only to a restricted group of specialists familiar with the nature of the conflict in the given research area. YET is typically analyzed as a concessive conjunct expressing that the post-conjunct section (Y) is surprising in view of the pre-conjunct section (X). It basically functions to mark seeming inconsistency between X and Y and to emphasize the suprising, puzzling or problematic nature of this inconsistency.

EXAMPLE: ‘One is impressed by the remarkable stability of the DNA molecule, an essential factor if species are to remain unchanged over many generations. YET the DNA must exist in a milieu of deleterious factors that can alter or destroy its genetic message.’

YET is particularly useful for setting up problems which are to be solved in the text that follows.

YET can signal exception to a generalization. EXAMPLE: ‘Smallpox has been eradicated in civilized countries. YET there were two cases reported from Birmingham.’

YET can be used also as a marker of frustration. EXAMPLE: ‘More sophisticated equipment could solve the given problem, YET it is not available in our country.’

STILL, another dissonant conjunct, is rather problem-loaded for NNS. Yet it helps to know that STILL is always related to an ongoing process of argumentation, evaluation or deliberation in the discourse. It is tied to the communicative purposes of the author. STILL is associated with a description of a state of affairs that remains constant in spite of threatened or expected change. EXAMPLE: ‘HPG was used in treating infertility for many years (X) when it was found to induce Jacob-Creuzfeld's disease (Y). STILL doctors went on using it, particularly in Australia’ (Z). The relationship is: X Y still Z. Y runs counter X, Z counter Y but is consistent with X.

STILL can also mark conflicting evidence bearing on a conclusion under consideration. EXAMPLE: ‘On the basis of the patient's history, the GP diagnosed MS. Still the symptoms are too general to justify the diagnosis.’ The understanding of ‘still’ in this hypothetical example is tied to the communicative purposes of the writer. The dissonance marked by ‘still’ is related to the ungoing process of argumentation and evaluation.

YET and STILL, two rather important signals with a gamut of implicit meanings, are practically absent in NNS manuscripts. Understanding the use of a particular dissonant conjunct in a particular text rests on understanding the nature of the conflict that the conjunct is marking. The sense in which Y can be in conflict with X varies considerably and the appropriateness of particular conjuncts is related to these various senses.

ON THE CONTRARY follows a negative statement with which it expresses non-dissonance. EXAMPLE: ‘The method is not restricted to special circumstances, ON THE CONTRARY, it is used routinely and on a massive scale.’

ON THE OTHER HAND merely marks dissimilarity, mostly without any suggestion of conflict. It assigns nearly the same logical status to X and Y and so their position may be reversed with little change in the logical relationship between them.

The same applies to NEVERTHELESS and NONETHELESS, best described as cancelling a plausible inference. Rather than signaling an expected causal relationship, they signal denial of an expected negative correlation. On the basis of X, one would expect something other than what is presented in Y.

DESPITE THIS and IN SPITE OF THIS are appropriate in many of the same contexts as the former two conjuncts, nevertheless and nonetheless. They are limited to the denial of a naturally expectable consequence of circumstances described in X.

The dissonant conjunct EVEN SO is rarely used by NNS. It can mark variance between X and Y both in terms of the world outside the text and in terms of the line of argument developed within the text. In contrast with the symmetrical relationship marked by ‘yet’ and ‘on the other hand’, ‘even so’ signals an asymmetrical relationship. It does not give equal logical status to X and Y, indicating that Y is more forceful than X. EXAMPLE: ‘The above discussed drugs can help diabetic patients in many ways. EVEN SO, their adverse effects would act too visciously to justify long-term administration.’

Several conjuncts, though similar in function, are not freely substitutable for each other with the same effect. HOWEVER may be considered an exception as it is most generally contrastive. It can probably be safely substituted for ‘yet, even so, still, at the same time, on the other hand, nevertheless, nonetheless’. And yet, a shade of relevant meaning may get lost by any substitution.

The appropriateness of dissonant conjuncts depends mostly on the ongoing communicative process. They are practically inseparable from the larger discourse contexts in which they appear. Thus the above uncontextualized presentation, which has necessarily ignored several communicative uses to which dissonant conjuncts can be put, should be considered with caution. A reliable understanding of this rather complicated problem area can only be achieved by studying their application in actual discourse.

All the devices of the English modality system are important means of negotiability of propositions in science reporting. They allow to make claims and criticize the claims of other authors without threatening their face and thus the face of the scientific community. The intercultural form of awareness in the native – NNS paradigm should be aimed at pragmalinguistic and sociocultural adequacy in producing academic discourse. Mastering of the appropriate use of modality devices contributes considerably to the mark of professional communicative competence.

### 5. Assessment of NNS science reporting by peers

Quality judgement of a paper submitted for publication in a scientific journal is exerted by editors and peer reviewers. As indicated in the present paper, there may be cross-culturally different perceptions of appropriate language behavior. The evaluative process of making decisions about content and style is guided by the writer's internal standards, which may vary from one discourse community to another. When the writer's and referee's internal standards are not congruent, the paper may be found defective.

The question of reliability of peer reviews (PR) is a rather controversial issue (Lock, 1991). Overall, peer review has worked efficiently and has survived over three centuries. Uncritical acceptance of the claims of others is considered a failure to meet rationality requirements imposed on genuine knowledge. Vigilance towards the source may however become tainted with mistrust or even prejudice and unduly affect acceptance or rejection of communicated information (Sperber *et al.*,^2010^). Judgement of competence and trustworthiness is in some instances formed on the basis of very limited evidence. Editors and reviewers often discriminate by judging colleagues on their work address, not always a neutral piece of information when assessing a paper. Status preferences, intolerance against non-native writers and systematic bias as to the reputation of the author's institution have been repeatedly pointed out (Peters & Ceci, 1982; Gannon, 2007).

In contrast to English journal articles and monographs characterized by a high degree of politeness making personal attacks inadmissible, the anonymous PR has different politeness pragmatics. The evaluative feature of the PR involves inherently face threatening acts, which are not always redressed by politeness devices to mitigate negative judgements and commands.

An analysis of peer reviews of 80 NNS manuscripts (Kourilova, 1998) highlighted both research-related and culture-specific shortcomings as judged by the reviewer.

Omission of data, particularly supportive evidence, was the most frequent target of criticism. Unjustified conclusions, frequently due to the lack of ability to show in English that the argument is well thought out, seem to be the most severe charge,

Other areas of criticism in the analyzed series concerned condensation techniques, cohesion devices, and very importantly the culture-determined use of modality. The charges involved excessive assertiveness, unhedged, unmodified statements, inadequate use of the article signaling unjustified generalization, as well as the inappropriate use of tenses failing to leave the claim open to negotiation by the scientific community.

In the whole series there were 671 instances of criticism, *i.e.* 8.39 per review. Of these 121 were classified as hedged criticism, yet 550 instances presented blunt criticism, including 24 cases of irony. There were 160 unmitigated commands (2.0 per review) and 64 commands were mitigated to suggestions.

In the analyzed series, criticism, though often blunt and caustic, was rarely unsubstantiated. Nevertheless, PR may involve misjudgment and bias, particularly towards the NNS’ language and his/her institution. There are variations in the quality of feedback peer reviews are able to provide about the language and writing – advice that does not always help to make the writing better. Out of 59 instances criticizing the language itself, 25 were unspecified (*e.g.* ‘The English needs improvement’). Not only native speakers act as reviewers, as documented by two instances of wrong spelling, *i.e.* failing to capitalize: 'The english needs improvement.’ The bias to the author's institution and country of origin may be quite strong.

## Conclusions

The main aim of the paper was to assist NNS in processing English scientific discourse that would not violate the reader's expectations by interfering with ready comprehension and socio-cultural conventions.

Areas of frequent violations of English scientific discourse management treated in the paper included lack of proficiency in handling the development of the author's argument and clear logical linking of propositions, as well as inadequate evidence supporting the main propositions. An important target of criticism concerned the culture of assessing own results and evaluating rival claims. NNS appear to be less aware of subtle degrees of truth commitment and of potentially face threatening acts than their English, and particularly British counterparts.

The shortcomings are frequently caused by the NNS's pragmalinguistic and sociocultural failure to master the organizational conventions of discourse processing and the broad repertoire of devices of the English modality system. These phenomena fall within the flexible area of hidden grammar, absorbed subconsciously by native speakers, yet very difficult to teach and learn by NN users of the language. The aim of the present paper would be achieved if it proved useful in helping researchers to make adequate syntactic and lexical choices so as to master semantic transparency and observe sociocultural conventions in producing English scientific discourse.
